# A cross-sectional survey of experts’ opinions about the relative effectiveness of tobacco control strategies for the general population versus disadvantaged groups: what do we choose in the absence of evidence?

**DOI:** 10.1186/1471-2458-13-1144

**Published:** 2013-12-08

**Authors:** Christine L Paul, Heidi Turon, Billie Bonevski, Jamie Bryant, Patrick McElduff

**Affiliations:** 1 Priority Research Centre for Health Behaviour (PRCHB), School of Medicine and Public Health, University of Newcastle, Newcastle, Australia; 2 Clinical Research Design, IT and Statistical Support (CReDITSS) unit, University of Newcastle, Newcastle, Australia; 3 Hunter Medical Research Institute (HMRI), Newcastle, Australia; 4 Public Health /HBRG, W4, HMRI Building, University of Newcastle, Callaghan, Newcastle, NSW 2308, Australia

**Keywords:** Smoking cessation, Vulnerable populations, Expert opinion, Socioeconomic status, Indigenous health, Mental illness, Low income population

## Abstract

**Background:**

There is a clear disparity in smoking rates according to social disadvantage. In the absence of sufficiently robust data regarding effective strategies for reducing smoking prevalence in disadvantaged populations, understanding the views of tobacco control experts can assist with funding decisions and research agendas.

**Methods:**

A web-based cross-sectional survey was conducted with 192 respondents (response rate 65%) sampled from the Australian and New Zealand Tobacco Control Contacts list and a literature search. Respondents were asked to indicate whether a number of tobacco control strategies were perceived to be effective for each of: the general population; Aboriginal and Torres Strait Islander people; those with a low income; and people with a mental illness.

**Results:**

A high proportion of respondents indicated that mass media and increased tobacco taxation (84% and 89% respectively) were effective for the general population. Significantly lower proportions reported these two strategies were effective for sub-populations, particularly Aboriginal and Torres Strait Islanders (58% and 63% respectively, p’s < .0001). Subsidised medication was the only strategy associated with a greater proportion of respondents perceiving it to be effective in disadvantaged sub-populations compared to the general population. Tailored quit programs and culturally relevant programs were nominated as additional effective strategies for disadvantaged populations.

**Conclusions:**

Views about subsidised medications in particular, suggest the need for robust cost-effectiveness data relevant to disadvantaged groups to avoid wastage of scarce tobacco control resources. Strategies perceived to be effective for disadvantaged populations such as tailored or culturally relevant programs require rigorous evaluation so that potential adoption of these approaches is evidence-based.

## Background

Despite long-standing tobacco control efforts, a persistent socioeconomic gradient in smoking prevalence exists in a number of western countries [1-3]. In Australia, markedly higher smoking rates are found among highly disadvantaged groups including Aboriginal and Torres Strait Islander people (38%), individuals who are unemployed or with a low income (25-30%) and people with a mental illness (36%) compared with the general population (15.1%) [4-6]. There is also evidence of a prevalence gradient within disadvantaged groups. For example, Aboriginal and Torres Strait Islanders typically experience several types of disadvantage, with research showing increasing smoking prevalence as a function of decreasing household income [7]. Effective tobacco control initiatives which address smoking in all socioeconomic groups are critical to addressing this social disparity.

There is growing debate about how to best address disparities in tobacco use and subsequent health outcomes [8-10]. Behavioural interventions have shown promise amongst some disadvantaged groups although the evidence is mixed [11]. There remains a lack of robust evidence about the effectiveness of a number of tobacco control strategies for disadvantaged groups [11,12]. Evidence on the relative effectiveness of major strategies such as mass media and telephone support does not address the full range of disadvantaged groups, so is insufficient for decision making and policy development. For example, studies of the effectiveness of anti-tobacco mass media campaigns across socioeconomic groups commonly omit the highly disadvantaged [12]. Inter-group comparisons of the relative effectiveness of population-based strategies have focussed on gender, age and some racial groups rather than Indigenous status, poverty or mental health [13].

The lack of methodologically rigorous evidence about the most effective tobacco control strategies for redressing the socioeconomic gradient in smoking rates can impede decision making, funding and agenda setting. Consequently, decisions can be easily influenced by individual opinion. In the absence of robust trial data, representative data on the views of relevant stakeholders can be helpful in guiding decision making, setting a research agenda or simply understanding where resources are likely to be channelled in the absence of evidence. While little is known about the views of experts, views about effective tobacco control strategies have been sought from disadvantaged smokers. For example, remote indigenous community members indicated brief advice and pharmaceutical quitting aids were perceived as important and effective, as was introduction of smoke free areas [14]. In contrast, there were conflicting views on Quit programs and tobacco taxation increases. Qualitative and quantitative studies of disadvantaged groups attending social and community service organisations have identified financial or material incentives and subsidised pharmacotherapies as popular choices for cessation support [15,16]. Understanding the views of the tobacco control community is important not only to assist with decision making, but to understand the perceptions guiding current decision making, and identifying targets for strategic research funding.

This study aimed to explore the views of a sample of Australian and New Zealand tobacco control advocates, researchers and workers regarding:

1. Perceptions of the effectiveness of i) *Population-level strategies* such as mass media campaigns, taxation increases, limits on expenditure of government payments; and ii) *Cessation support* such as telephone or SMS cessation support, subsidised pharmacotherapies and web-based cessation programs; for the general population and for each of three socially disadvantaged groups: Aboriginal and Torres Strait Islander people, those with a low income and people with a mental illness.

2. Additional strategies perceived to be effective for these disadvantaged groups.

## Methods

### Design

An online cross-sectional survey was conducted in September and October, 2011.

### Sample

Two hundred and ninety two people listed on the 2011 Australian and New Zealand Tobacco Control Contact List were invited to participate in an online survey. The Australian and New Zealand Tobacco Control Contact List is a directory of individuals working in tobacco-related research, advocacy, tobacco control policy and program delivery. We identified additional relevant contacts by conducting a PubMed search using the following search parameters (Australia and/or New Zealand) and (Smoking and/or tobacco control) for articles published between 1/1/2008 and 31/12/2010. We included 51 additional individuals with Australian or New Zealand affiliations who were listed as one of the first three authors on at least three relevant publications, and were not already listed on the Australian and New Zealand Tobacco Control Contact List.

### Procedure

Participants were invited to complete an anonymous web-based survey hosted by Survey Monkey™. Potential respondents were sent an email explaining the purpose of the survey, how they were chosen to participate, and inviting them to complete the survey by clicking on a weblink. All non-respondents received a reminder email one week after the initial invitation, and an additional reminder telephone call (or email if not contactable by telephone) 7-10 days after the reminder email was sent. Ethical approval was obtained from the University of Newcastle Human Research Ethics Committee.

### Web survey items

The survey contained 11 items exploring views about the degree to which tobacco control efforts should focus on whole population approaches versus focusing on disadvantaged groups. Data from survey items on the resourcing of tobacco control mass-media campaigns and research priorities are reported elsewhere [10]. Data for four items on the perceived relative effectiveness of tobacco control strategies are reported here. For each group of interest (General population, Aboriginal and Torres Strait Islander People, People with a Low Income and People with a Mental Illness), respondents were asked “Which of the following strategies do you think will be effective for reducing smoking prevalence?”. Respondents could select from eight response options: ‘Mass media campaigns’, ‘Increased taxes on tobacco’, ‘Ensuring government payments (e.g. Centrelink) cannot be spent on tobacco’, ‘Telephone or SMS support (e.g. Quitlines)’, ‘Web-based approaches’, ‘Subsidised medications (e.g. Nicotine patches)’, ‘None of the above’ or ‘Other’-specify. Respondents could select more than one strategy.

### Analysis

Descriptive statistics including means and medians were used to explore the data. Although most of the data were normally distributed, in a few instances responses were skewed. Logistic regression within a Generalised Estimating Equation (GEE) framework was used to make comparisons regarding the proportions of respondents replying in the positive for effectiveness to each particular strategy. P values reported from the GEE are from a test of the comparison of ‘yes’ responses to each population compared to a ‘yes’ response to the general population.

## Results

### Sample

The 343 invitations resulted in 192 completed surveys (49 were ineligible due to invalid email, on leave, changed roles), giving a response rate of 65% (see Table 1 for demographic characteristics). Overall, our respondents were representative of all those invited to participate with regard to gender and residence; 61% of respondents were female (62% of those invited were female), 25% of respondents were from NSW (23% of those invited), 21% were from Victoria (20% of those invited), and 20% were from New Zealand (18% of those invited).

**Table 1:** Demographic characteristics of respondents

	N*	**% of respondents**
***Gender***		
Female	110	61
***Residence***		
NSW	46	25
Vic	38	21
New Zealand	36	20
Other Australian states	61	34
***Work role***		
Researcher	56	31
Advocacy/Policy Work	47	26
Service or program management	34	19
Service or program delivery	26	14
Other	18	10
***Proportion of work role focused on disadvantaged groups***		
Half or less	94	52
More than half	44	24
All	21	12
Other	22	12
***Experience in tobacco control***		
1-5 years	56	31
5-10 years	58	32
11-20 years	40	22
Other	27	15

*Note: Ns do not sum to 192 due to missing demographic data.

### Perceived effectiveness of specific strategies for each group of interest

#### Population-Level measures

**Figure 1:**
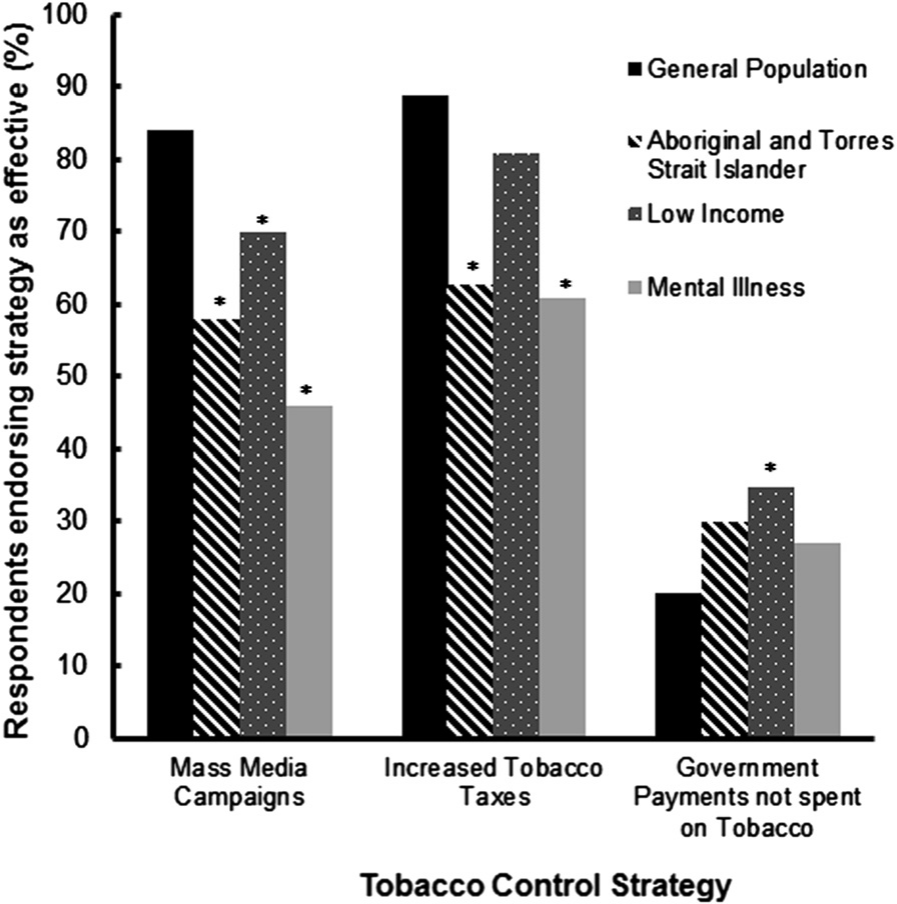
Proportion of respondents endorsing population-level tobacco control strategies as effective for each population (* p <0.0001 compared to the general population).

The perceived effectiveness of each population-level tobacco control strategy for each group is shown in Figure 1. Comparisons of the perceived effectiveness of mass media campaigns for the four population groups indicated that mass media was perceived by significantly more of the respondents to be an effective tobacco control strategy for the general population (84% of respondents) than for low income groups (70%, p < .0001), Aboriginal and Torres Strait Islander people (58%, p < .0001), and people with a mental illness (46%, p < .0001). Significantly fewer respondents perceived increased taxes to be effective for Aboriginal and Torres Strait Islander people (63%, p < .0001), people with a mental illness (61%, p < .0001) and low income groups (81%, p < .01) than for the general population (89%). Increased tax on tobacco was also the only strategy in which perceptions of effectiveness varied significantly with respondent experience, but only for some groups. The proportion of respondents who endorsed increased tobacco taxes as effective increased with the number of years working in tobacco control (p < .005 for Aboriginal and Torres Strait Islander peoples, and p < .05 for people with a mental illness). Significantly higher proportions of respondents perceived that ensuring government payments could not be spent on tobacco would be effective with people with a mental illness (27%, p < .05), Aboriginal and Torres Strait Islander people (30%, p < .005), and low income groups (35%, p < .0001), compared to the general population (20%).

#### Individual-Level Measures

**Figure 2:**
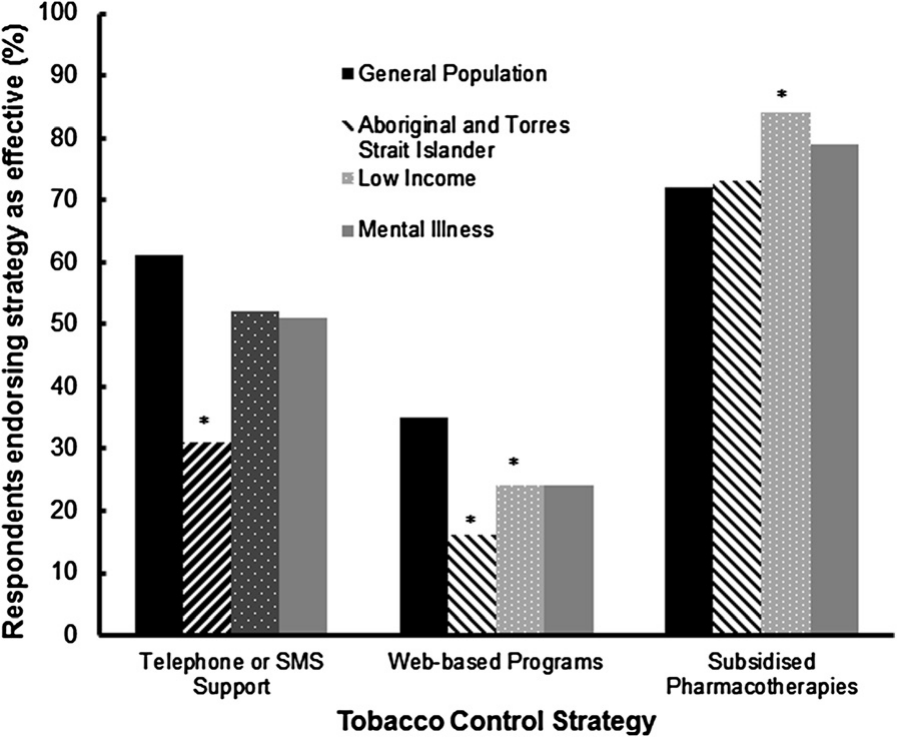
Proportion of respondents endorsing individual-level tobacco control strategies as effective for each population (* p <0.0001 compared to the general population).

The perceived effectiveness of each individual-level tobacco control strategy for each group is shown in Figure 2. Telephone or SMS cessation support was perceived by significantly more respondents to be an effective tobacco control strategy for the general population (61% of respondents) than for low income groups (52%, p < .005), people with a mental illness (51%, p < .005) and Aboriginal and Torres Strait Islander people (31%, p < .0001). Significantly more respondents perceived web-based cessation support to be an effective tobacco control strategy for the general population (35% of respondents) than for low income groups (24%, p < .0001), people with a mental illness (24%, p < .0005), and Aboriginal and Torres Strait Islander people (16%, p < .0001). Significantly more respondents perceived subsidised pharmacotherapies to be more effective for low income groups (84%, p < .0001) than for the general population (72%). There was no difference between the proportion of respondents reporting subsidised medications were effective for the general population than for people with a mental illness (79%, p > .05) and Aboriginal and Torres Strait Islander people (73%, p > .05).

### Other strategies perceived to be effective for specific populations

Fifty-five respondents identified additional strategies they believed may be successful in reducing smoking prevalence in the general population. Additional strategies nominated by more than one respondent as potentially effective for each of the sub-populations are reported in Table 2. An extension on smoking bans in public areas was the most commonly proposed additional strategy for the general population. One hundred and seven respondents identified additional strategies to reduce smoking prevalence among Aboriginal and Torres Strait Islander peoples, with community and culturally-based elements featuring in the nominations. A total of 66 respondents nominated additional strategies for reducing smoking prevalence in low income populations including proposed extensions on smoke free environments (n = 15) and tailored quit programs (n = 8). Ninety-six respondents suggested alternative strategies to reduce smoking prevalence among people with a mental illness including tailored quit programs (n = 12) and intensive support from health professionals and services (n = 10).

**Table 2:** Additional tobacco control strategies perceived to be effective for different population groups

**Additional Strategies**	**N**
***General Population***	
Extension on smoking bans in public areas	24
Advertising restrictions/plain packaging	12
Reducing locations of sale	6
Increased legislation	5
***Aboriginal and Torres Strait Islander peoples***	
Community based strategies	25
Involvement of elders and others in strategy development and education	17
Tailored quit programs	17
Using culturally relevant information to inform strategies	10
Targeted media campaigns	8
Restricting availability of tobacco products	7
Interventions involving health professionals	7
***Low income populations***	
Extension on smoke free environments	15
Tailored quit programs	8
Advice and support from health professionals	7
Restricting availability of tobacco products	6
***People with a mental illness***	
Tailored quit programs	12
Intensive support from health professionals and services	10
Extension on smoke free environments	9
Face-to-face interventions	8

## Discussion

This survey of key tobacco control stakeholders found high levels of perceived effectiveness of population-based strategies including mass media campaigns and increased taxes for the general population. Fewer respondents were convinced that these strategies would be effective for disadvantaged sub populations with high smoking rates. In general, the number of years working in tobacco control did not affect perceptions of strategy effectiveness. The one exception to this was that those with more experience were more likely to rate increased taxes on tobacco as an effective strategy for Aboriginal and Torres Strait Islander peoples and people with a mental illness, compared to those who had not been working in tobacco control for as long.

Approximately half of the sample considered mass media to be effective for Aboriginal and Torres Strait Islander peoples and people with a mental illness, significantly lower than the 84% perceiving an effect for the general population. It is not possible to judge whether respondents believed this was due to the medium (e.g. television) or the message (e.g. lack of culturally relevant actors or issues). However, the data suggest that one of Australia’s largest tobacco investments is not therefore, considered to be an avenue for reducing the social disparity in smoking rates. While there are data to suggest that mass media can be effective for lower income groups [17], the issue of differential effectiveness of mass media for very disadvantaged groups has not been adequately addressed in the literature [12]. Recent Australian campaigns aimed at delivering culturally-appropriate mass media tobacco control messages for Aboriginal people require sound evaluation and dissemination of data to ensure the tobacco control community is aware of developments in this area [18,19].

Similarly, widely available cessation strategies such as telephone, SMS, and web-based programs were perceived by a much lower proportion of respondents to be effective for disadvantaged groups (16%-52%) than for the general population (35%-61%), most notably for Aboriginal and Torres Strait Islander people. A survey of low-income clients accessing social and community support organisations, also found low levels of support for receiving smoking cessation advice via Quitlines or SMS services [16]. Given that telephone, SMS and web-based support are the most promoted and available forms of cessation support in Australia, there is a need for robust research demonstrating whether very low levels of relative effectiveness are the case for such groups compared to the general population. If this is the case, it is necessary to develop effective strategies for improving the reach, efficacy and effectiveness of cessation support strategies for groups with particularly high smoking rates. One Australian trial of the telephone support service (Quitline) with a disadvantaged sample found no evidence of effectiveness at 12 months follow-up [20]. More recent evidence also suggests lower levels of engagement and less effectiveness for Aboriginal and Torres Strait Islander people accessing the Quitline [21], a finding which is echoed by a survey of Indigenous community members and health workers, suggesting unmodified Quit programs may lack appropriateness in such settings [14].

In contrast, ensuring government payments are not spent on tobacco was perceived by more respondents to be effective for each disadvantaged group than for the general population. However, the levels of endorsement were low (20%-35%), suggesting this was not an approach that would be broadly supported by the tobacco control community. Subsidised medication was the sole instance where the proportion who perceived this strategy to be effective was high (72-84% of respondents) for all population groups, with higher proportions of endorsement for some disadvantaged groups than for the general population. This suggests the respondents perceive addiction or finances (or both) to be of major importance in reducing smoking in disadvantaged groups. Given the high cost of this approach and concern about the real-world effectiveness of pharmacotherapies [22,23], careful testing of the relative cost-effectiveness of medications for disadvantaged versus general population groups is needed.

Also of interest are the additional strategies nominated by respondents as potentially effective as an indication of what key stakeholders may endorse or advocate. Increased restrictions on smoking in public areas were nominated as likely to be effective for all groups other than for Aboriginal and Torres Strait Islander people, while tailored quit programs were suggested for each disadvantaged group. Tailoring of cessation programs to disadvantaged populations has intuitive value, particularly for Indigenous groups given the unique social and cultural factors which influence smoking behaviour in this population [24]. Evidence regarding tailored cessation programs in disadvantaged populations is limited, and has been the subject of some debate particularly in relation to tailoring for Indigenous populations. One review advocated for the development of tailored and targeted approaches to smoking cessation [25], while another review argued that not all smoking cessation programs need to be culturally adapted to be effective [26]. However, both reviews only included a small number of studies, suggesting further research is necessary to fully inform the tobacco control community on the value of tailored programs. In relation to people with a low income or mental illness, programs delivered by health care providers and service providers were nominated. While there is a growing evidence base in this field [27-31], this work is in its infancy. The dissemination of effective approaches should also be studied, given the identified challenges in achieving high rates of cessation advice in primary care [32]. Strategies nominated as likely to be effective for Aboriginal and Torres Strait Islander people suggest community-based, culturally relevant approaches that involve elders and other community leaders are preferred. Given the weakness of the evidence-base around culturally-relevant programs [33], it is important to establish robust science around the implementation of cultural relevance to ensure tobacco control programs for Aboriginal and Torres Strait Islander people provide the greatest possible benefit.

When considering the evidence for the effectiveness of strategies for Aboriginal and Torres Strait Islander people, it is important to consider the implications of this for the Indigenous population of New Zealand. The Maori population makes up a larger proportion of the New Zealand population (approximately 15%) [34] compared to Aboriginal and Torres Strait Islanders, who make up approximately 2.5% of the Australian population [35]. However, both Indigenous populations experience many health disparities compared to the non-Indigenous population including a significantly lower life expectancy, and a higher smoking prevalence [35,36]. Therefore it remains important to continue research into effective strategies for promoting smoking abstinence in these populations, and where possible, identify approaches that are most effective and acceptable for each group.

The interpretation of these findings should take into account some limitations. Firstly, although the response rate is acceptable, it does not preclude response bias. It is possible that those who did not respond may have expressed different views to those of the respondents. Those working with disadvantaged groups may have been more likely to respond than those without such involvement, and so may have stronger views about the needs of such groups in relation to tobacco control strategies. Furthermore, it is acknowledged that this survey was conducted at a time in which the Indigenous tobacco control workforce was undergoing substantial change and growth. As such, some of these workers may not have been on the contact list, and therefore would not have been invited to complete the survey.

It is also likely that the sample was heterogeneous in terms of familiarity with data about the effectiveness of the various tobacco control strategies. While the heterogeneity of the sample may be considered a limitation, it may also be considered a study strength. It should also be noted that no explanation was provided regarding definitions of the sub populations. This may be a concern in relation to interpretation of the term ‘low income’ which can be interpreted as less than average income or below the poverty line which are quite different groups, the latter of which is not well-reflected in studies of the effectiveness of increased taxes on smoking cessation [37]. These groups also have significantly different smoking prevalence rates- 24.6% in the most disadvantaged quintile [4], compared to 61% among individuals with multiple forms of disadvantage accessing social and community service organisations [16]. As the survey did not provide respondents with a definition of low income, it may be that their responses reflect wide variations in definitions of low income groups.

## Conclusions

In order to produce an evidence base for tobacco control policy and service delivery it is important to understand and address the views of the tobacco control community. Given the lack of robust evidence about intervention effectiveness for more disadvantaged smokers [11], a sizeable research effort is needed to reduced smoking rates among disadvantaged groups. The views of those engaged in tobacco control in Australia and New Zealand suggest the need for a targeted research agenda to establish whether current tobacco control strategies such as mass media and telephone support can become substantially more effective for disadvantaged groups than is currently perceived to be the case. Given these views may be at odds with the emphasis on mass media approaches within the Australian National Tobacco Control Strategy, there may be a need for closer engagement between policy makers and those working with disadvantaged groups. The popularity of subsidised medications requires robust cost-effectiveness data to determine population-specific cost-effectiveness. Other strategies likely to be popular for reducing smoking disparities such as tailored programs and culturally relevant programs also require rigorous evaluation.

## Competing interests

The authors declare that they have no competing interests.

## Authors’ contributions

All authors conceptualised the study and contributed to the design of the project. CP and HT were involved in data collection, HT and PM were involved in data analysis and all authors contributed to data interpretation and drafting and revising the manuscript. All authors read and approved the final manuscript.

## Pre-publication history

The pre-publication history for this paper can be accessed here:

http://www.biomedcentral.com/1471-2458/13/1144/prepub
